# Does job control contribute to differences in physician-certified sickness absence across office concepts? A mediation analysis in a nationally representative sample

**DOI:** 10.5271/sjweh.4167

**Published:** 2024-09-01

**Authors:** Randi Hovden Borge, Håkon A Johannessen, Knut Inge Fostervold, Morten Birkeland Nielsen

**Affiliations:** 1 Research group for Work Psychology and Physiology, National Institute of Occupational Health, Oslo, Norway.; 2 Department of Psychology, University of Oslo, Oslo, Norway.; 3 Department of Psychosocial Science, University of Bergen, Bergen, Norway.

**Keywords:** absenteeism, autonomy, non-territorial office, office work, open office, privacy

## Abstract

**Objectives:**

Several studies have found higher sickness absence in shared and open workspaces than in private offices, but little is known about why these differences occur. We propose and test job control as a potential mechanism underlying observed differences in the risk of physician-certified sickness absence between private offices and shared and open workspaces.

**Methods:**

We conducted a counterfactual mediation analysis using observational survey data from a nationally representative sample of Norwegian employees merged with prospective data from national registries (N=5512). The registry data included information about whether participants had any physician-certified sickness absence the year following the survey. Models were adjusted for age, sex, education level, occupation group, executive/leadership responsibility, and time spent on office work.

**Results:**

We found significantly higher sickness absence risk in conventional [risk ratio (RR) 1.12, 95% confidence interval (CI) 1.01‒1.25] and non-territorial (RR 1.20, 95% 1.04‒1.37) open-plan and non-territorial shared-room offices (RR 1.29, 95% CI 1.13‒1.48) compared to private offices. Natural indirect effects due to job control were statistically significant in all contrasts and accounted for 19–34% of total effects depending on contrast.

**Conclusions:**

Findings were in line with hypothesized relationships and suggest that job control may be a mechanism underlying observed differences in sickness absence across office concepts. Future studies should continue to explore potential mechanisms linking shared and open workspaces to higher sickness absence and other unfavorable outcomes in the workplace, particularly with study designs that provide stronger basis for causal inference.

Shared and open workspaces are widespread in contemporary working life. Varying in size and functionality, common characteristics are less office space per employee and fewer physical barriers between workstations. While this allows organizations to save space, potentially reducing operating costs and climate footprint, conceptual (1, 2) and empirical (3, 4) work suggest unfavorable employee and organizational outcomes compared to private offices. One such outcome is sickness absence (5), with previous studies indicating both a higher probability of sickness absence (6–8) and more sickness absence days (9, 10) in shared and open workspaces than private offices. As sickness absence is costly for both individuals, organizations, and welfare states (11), it is important to understand why these differences occur (eg, to identify targets for evidence-based intervention). Yet, rather than empirically testing underlying mechanisms, past empirical work has focused almost exclusively on identifying and quantifying differences in sickness absence and other health outcomes (12). As the first empirical study to test a potential mediator of associations between office concepts and sickness absence, we examined (i) differences in risk of physician-certified sickness absence across office concepts in a nationally representative sample and (ii) how much of these differences are attributable to differences in job control [ie, employees’ perceived control over timing, methods, and decisions at work (13)].

Influential theories in occupational health [eg, the job demands resources model (14) and the demands-control-(support)-model (15)] and ample empirical evidence (16) identify job control as a central psychosocial determinant of employee health and well-being, including sickness absence (17). A salient feature differing across office concepts is psychological privacy (2–4), that is, the means available to control inputs from the environment to oneself (eg, distractions) and outputs from oneself to the environment (eg, confidentiality) (18). According to privacy regulation theory (18–20), psychological privacy constitutes a central mechanism for exercising secondary control over outcomes, as pacing and regulating social interaction enable us to set favorable conditions for goal-directed behavior (19) such as the completion of primary work tasks.

Synthesized evidence indicating more distractions and less control over workspace in shared and open workspaces than in private offices (3, 4), suggests office concepts differ in the means employees can use to exercise this condition-setting control, potentially affecting experiences of control over timing, methods, and decisions at work. Private offices represent a “flexible barrier” (18) with physical and functional features, such as walls and doors, that enable employees to adapt the social environment to fit specific work tasks (eg, concentration tasks, phone conversations) and individual needs. In contrast, shared and open workspaces lack the same means to control psychological privacy and, thereby, also the work process. For instance, they limit discretionary power to choose to work in private when needed (2). This may influence control over how and when to perform certain work tasks (eg, postponing phone conversations when no meeting rooms are available), which are key aspects of job control (13, 16). It may also create feelings of being overlooked and overheard (3), potentially triggering self-monitoring behaviors (eg, adjusting behavior when others are present). Shared and open workspaces also limit the opportunity to create settings that facilitate uninterrupted work. This implies less autonomy, as supervisors and co-workers are more likely to “interfere with or infringe upon an employee’s discretion and freedom to work” (21), and more work disruptions due to unpredictable and uncontrollable environmental stimuli (2), including both intended and unintended interruptions from colleagues (3, 4).

Empirical support for a link between office concepts and job control can be found in a recent longitudinal study where job control was lower among employees in shared-room and open-plan offices and control over work pace increased for those who moved to private offices (22). Similar relationships have been observed in two previous cross-sectional studies (23, 24). While few other studies have linked office concepts to job control empirically, several scholars have proposed that such a link is plausible (25–28).

Non-territorial offices where several employees share a number of unassigned workstations (eg, hot desking, activity-based offices) are increasingly prevalent in contemporary office work (29), thus suggesting that non-territoriality is a relevant factor to consider (12, 27, 30). Although studies indicate that few employees seem to switch workstations (31, 32), conceptual arguments and some empirical findings suggest that employees’ control over workspace may be different in some types of non-territorial offices (eg, activity-based offices) compared to conventional ones, because employees in the former may choose where to sit (29). Empirical findings regarding how non-territorial offices relate to sickness absence are also inconsistent (6, 8, 10, 28). We therefore differentiated between shared and open workspaces with assigned (ie, conventional shared-room or open-plan offices) and unassigned workstations (ie, non-territorial shared-room or open-plan offices).

We hypothesized (figure 1) that employees in (i) conventional shared-room offices, (ii) conventional open-plan offices, (iii) non-territorial shared-room offices, and (iv) non-territorial open-plan offices would have higher risk of physician-certified sickness absence (hypothesis 1) and lower ratings of job control (hypothesis 2) than employees in private offices, and that job control would mediate differences in risk of physician-certified sickness absence between private offices and the other office categories (hypothesis 3).

**Figure 1:**
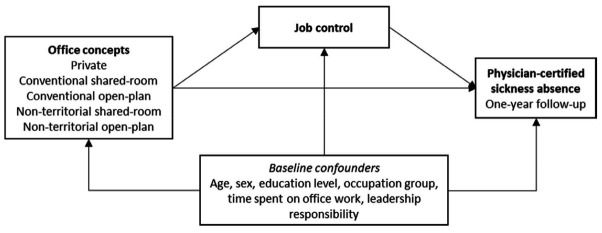
Model of hypothesised relationships between study variables. Baseline confounders included.

## Methods

### Sample and procedure

Data came from the Level of Living Survey on Working Conditions collected by personal telephone interviews between September 2016 and April 2017 (33). Potential participants received written information by mail prior to telephone contact. Of a gross sample of 20 272 individuals randomly drawn from the Norwegian population aged 17–67 years, 10 665 participated in the survey (53% response rate). Participation was based on informed consent. Eligible participants in the current study were employees in paid work who performed all or parts of their work in an office. Self-employed participants were not included in the study as they had not received questions about job control. After excluding participants with missing data on office concept, the final sample comprised 5512 participants.

### Study variables

*Office concept* was measured by a categorical variable based on both office layout (“do you work in your own office, shared-room office, or office landscape?”), number of office occupants (“how many people do you normally share an office with?”), and whether seating was fixed or free (“do you have a fixed workstation?”). The latter distinguished between shared-room or open-plan offices with assigned workstations (ie, conventional shared-room and open-plan offices) and offices with unassigned workstations shared among several employees (ie, non-territorial shared-room and open-plan offices). Several participants in shared-room offices reported sharing with three to nine people (N=689). The shared-room category therefore included shared-room offices with up to ten employees. Participants in shared-room offices who reported sharing with more than ten people, were placed in the open-plan categories (N=102). There were also some participants in open-plan offices who reported sharing with only one or two other people (N=121). These participants were placed in the shared-room categories. This resulted in five office categories (ie, private offices, conventional shared-room offices, conventional open-plan offices, non-territorial shared-room offices, non-territorial open-plan offices).

*Job control* was measured by four items rated on a scale from 1 (“to a very high extent”) to 5 (“to a very small extent”). One item addressed control over timing at work (“to what extent can you decide your own work pace?”), two items addressed control over methods at work (“to what extent can you decide which tasks you are given?” and “to what extent can you decide how to do your work?”), and one item addressed control over decisions at work (“to what extent can you influence decisions important to your work?”). Items addressing control over timing and decisions (ie, the first and the fourth) came from the General Nordic Questionnaire for Psychological and Social Factors at Work (34). Statistics Norway developed the items addressing control over methods. We reverse coded all items before combining them into a mean score with high scores representing high job control (α=0.75). Since the measure was not previously validated, we examined inter-item correlations and item-specific descriptive statistics across office concepts.

*Physician-certified sickness absence* was a dichotomous variable based on registry data from the Norwegian Labour and Welfare Administration (NAV) and indicated whether the participant had had physician-certified sickness absence during the year following the survey (ie, in 2017). This registry data had been merged with Statistics Norway survey data. Norwegian employees can self-certify their own sickness absence according to one of two regimes: they can either self-certify four times each year for ≤3 consecutive days if their employer follows the general rules for sickness absence or they can self-certify 24 days in total during a 12-month period if their employer is part of the agreement between the Norwegian Government and Social Partners on a More Inclusive Working Life (the IA Agreement). Sickness absence beyond this must be certified by a medical doctor. Employees are entitled to receive full pay from day one and for the whole first year of sickness absence.

### Statistical analyses

We performed all statistical analyses in R 4.2.1 (35). We conducted a counterfactual mediation analysis with the CMAverse package (36) using the regression-based approach with direct imputation of counterfactuals and bootstrapping with 1000 resamples to obtain standard errors and 95% confidence intervals (CI) (37). We specified one regression model for job control (linear regression) and one for sickness absence (log-linear regression), from which counterfactual effects (ie, average natural direct and indirect effects) were calculated. Log-linear regression were used for the outcome model to obtain risk ratios (RR), which is recommended in mediation analysis with binary outcomes that are not rare (37).

We used observational data to investigate hypotheses. A recent review on methodological considerations (12) suggests that employees in different office concepts differ on key demographic (eg, age, sex, education level) and occupational characteristics (eg, type of work, seniority). Similar characteristics may influence both perceptions of job control and the occurrence of sickness absence. We therefore included the following baseline covariates to adjust for these potential confounders: age (in years), sex (male/female), highest achieved education level (ie, primary/lower secondary school, upper secondary school, 1–4 years of university/college education, and ≥5 years of university/college education), main occupation group (according to the International Standard Classification of Occupations), a dichotomous variable for working time spent on office work (ie, ≤50% of the time), and a dichotomous variable for leadership/executive responsibility (ie, “does your position include leadership responsibility, so that other people work under your supervision, or is it otherwise an executive position?”). The latter two came from the survey data, and the rest came from national registries. Missing data were minimal and listwise deletion was therefore acceptable. This resulted in an analytical sample of 5412 participants.

Unlike conventional mediation analysis (eg, difference-in-coefficients, product-of-coefficients), counterfactual mediation analysis can incorporate exposure-mediator interaction (37). According to past conceptual work, job control may interact with demands in the workplace [ie, strain and buffer hypotheses (16)]. Although empirical support is limited (13), we compared regression models with and without an interaction term between office concept and job control to check for exposure-mediator interaction. Model comparison indicated similar model fit (likelihood ratio=6.44, P=0.276). We therefore proceeded with a two-way decomposition of total effects into average natural direct and indirect effects (37). The natural direct effect is the average effect of the exposure on the outcome with the mediator fixed at the level it would take in the reference category (eg, change in sickness absence risk when office concept is changed from private offices to conventional open-plan offices with job control fixed at the level observed in private offices). The natural indirect effect is the average effect of changing the mediator from the level it would take in the reference category to the level it would take in the exposure category with the exposure fixed at the reference category (eg, change in sickness absence risk when office concept is fixed at private offices and job control is changed from the level observed in private offices to the level observed in conventional open-plan offices).

## Results

Table 1 displays sample characteristics, overall and by office concept. Private offices were most common (43%), followed by conventional open-plan (23%), conventional shared-room (19%), non-territorial shared-room (9%), and non-territorial open-plan offices (7%).

**Table 1:** Sample characteristics, overall and by office concept. [SD=standard deviation.]

Variable		Overall (N=5512)			Private (N=2363)			Conventional shared-room (N=1022)			Conventional open-plan (N=1282)			Non-territorial shared-room (N=467)			Non-territorial open-plan (N=378)	
		Mean (SD)	N (%)		Mean (SD)	N (%)		Mean (SD)	N (%)		Mean (SD)	N (%)		Mean (SD)	N (%)		Mean (SD)	N (%)
Age		44.5 (11.7)			47.3 (10.8)			43.3 (11.8)			43.1 (11.2)			40.6 (13.2)			39.7 (12.3)	
Sex																		
	Male		2872 (52)			1368 (58)			492 (48)			674 (53)			174 (37)			164 (43)
	Female		2640 (48)			995 (42)			530 (52)			608 (47)			293 (63)			214 (57)
Education level																		
	Primary/lower secondary		665 (12)			284 (12)			146 (14)			100 (8)			83 (18)			52 (14)
	Upper secondary		1474 (27)			638 (27)			268 (26)			290 (23)			164 (35)			114 (30)
	University/college 1–4 years		2252 (41)			874 (37)			445 (44)			576 (46)			195 (42)			162 (43)
	University/college ≥5 years		1068 (20)			548 (23)			154 (15)			297 (24)			22 (4.7)			47 (13)
	Missing		53			19			9			19			3			3
Occupation group																		
	Managers		790 (14)			565 (24)			79 (8)			123 (10)			13 (2.8)			10 (3)
	Professionals		2388 (43)			921 (39)			478 (47)			662 (52)			172 (37)			155 (41)
	Technicians		1111 (20)			469 (20)			193 (19)			300 (23)			62 (13)			87 (23)
	Clerical support staff		348 (6)			130 (6)			83 (8)			86 (7)			18 (4)			31 (8)
	Services and sales workers		493 (9)			129 (6)			91 (9)			51 (4)			153 (33)			69 (18)
	Other occupations ^a^		382 (7)			149 (6)			98 (10)			60 (5)			49 (10)			26 (7)
Time spent on office work																		
	≥50%		3724 (68)			1896 (80)			550 (54)			1023 (80)			86 (18)			169 (45)
	<50%		1788 (32)			467 (20)			472 (46)			259 (20)			381 (82)			209 (55)
Leader/executive																		
	No		3313 (60)			1114 (47)			644 (63)			903 (71)			358 (77)			294 (78)
	Yes		2189 (40)			1243 (53)			377 (37)			377 (29)			108 (23)			84 (22)
	Missing		10			6			1			2			1			0
Job control (range 0–4)		2.53 (0.78)			2.74 (0.75)			2.44 (0.75)			2.46 (0.75)			2.20 (0.77)			2.13 (0.79)	
	Missing		30			13			6			5			5			1
Physician-certified sickness absence			1757 (32)			646 (27)			328 (32)			417 (33)			216 (46)			150 (40)

^a^ Other occupations = Groups 6, 7, 8, 9, and 0 merged into one category due to few participants reported doing office work.

### Counterfactual mediation analysis

**Figure 2:**
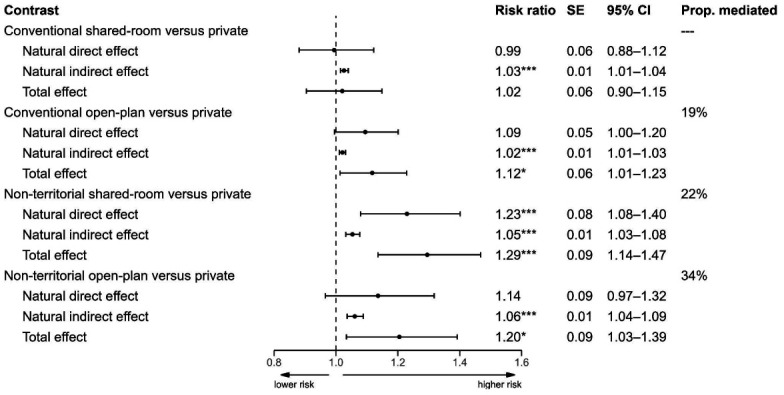
Total effects of office concepts on sickness absence risk decomposed into average natural direct and indirect effects through perceptions of job control. Risk ratios and 95% bootstrap confidence intervals. Estimates adjusted for age, sex, education level, occupation group, leadership/executive responsibility, and time spent on office work. *P<0.05, **P<0.01, ***P<0.001.

Figure 2 displays results from the counterfactual mediation analysis with total effects decomposed into average natural direct and indirect effects. In line with hypothesis 1, total effects indicated that, compared to private offices, the risk of physician-certified sickness absence was significantly higher in both conventional (RR 1.12, 95% CI 1.01‒1.25) and non-territorial (RR 1.20, 95% 1.04‒1.37) open-plan offices, as well as in non-territorial shared-room offices (RR 1.29, 95% CI 1.13‒1.48). The total effect of shared-room offices was not statistically significant and close to zero (RR 1.02, 95% CI 0.91‒1.14).

In line with hypothesis 2, results from the mediator regression model (table 2) indicated that job control was significantly lower in all office concepts compared to private offices. Coefficients ranged from -0.42 (95% CI -0.50‒ -0.34) in non-territorial open-plan offices to -0.14 (95% CI -0.19‒ -0.09) in conventional open-plan offices. Job control was significantly associated with lower risk of physician-certified sickness absence (RR 0.86, 95% CI 0.81‒0.93; table 2). In line with hypothesis 3, natural indirect effects (figure 2) indicated that job control significantly mediated parts of the differences in all contrasts. Proportions of total effects attributable to differences in job control were 19% for conventional open-plan offices (RR 1.02, 95% CI 1.01‒1.03), 34% for non-territorial open-plan offices (RR 1.06, 95% CI 1.04‒1.09), and 22% for non-territorial shared-room offices (RR 1.05, 95% CI 1.03‒1.08).

**Table 2:** Results from mediator and outcome regression models. [RR=risk ratio; CI=confidence interval.]

			Unadjusted model				Adjusted model^a^		
			*b*	RR	95% CI		*b*	RR	95% CI
Effects of office concepts on job control									
	Office concept								
		Private	(ref)		(ref)		(ref)		(ref)
		Conventional shared-room	-0.29 ***		-0.34‒-0.23		-0.18 ***		-0.23‒-0.12
		Conventional open-plan	-0.27 ***		-0.32‒-0.22		-0.14 ***		-0.19‒-0.09
		Non-territorial shared-room	-0.53 ***		-0.61‒-0.45		-0.36 ***		-0.44‒-0.28
		Non-territorial open-plan	-0.59 ***		-0.68‒-0.51		-0.42 ***		-0.50‒-0.34
Effects of office concepts and job control on sickness absence									
	Office concept								
		Private		(ref)	(ref)			(ref)	(ref)
		Conventional shared-room		1.09	0.95‒1.25			0.99	0.86‒1.14
		Conventional open-plan		1.12	0.99‒1.27			1.09	0.96‒1.24
		Non-territorial shared-room		1.52 ***	1.30‒1.78			1.23 *	1.03‒1.47
		Non-territorial open-plan		1.29 **	1.07‒1.54			1.14	0.94‒1.37
		Job control		0.81 ***	0.76‒0.86			0.87 ***	0.81‒0.93

^a^ Estimates adjusted for age, sex, education level, occupation group, leadership/executive responsibility, and time spent on office work. *P <0.05. **P <0.01. ***P<0.001.

### Additional analyses

Since main results indicated potential differences among the shared and open workspace categories along the territoriality dimension, we performed contrasts between non-territorial shared-room and open-plan offices and their conventional counterparts. Results indicated significantly higher risk of physician-certified sickness absence in non-territorial shared-room offices compared to conventional shared-room offices (RR 1.26, 95% CI 1.11‒1.45). Sickness absence risk was also elevated in non-territorial compared to conventional open-plan offices, but the effect was not statistically significant (RR 1.08, 95% CI 0.93‒1.25). Natural indirect effects were significant in both contrasts. They accounted for 52% of the total effect in the contrast between conventional and non-territorial open-plan offices (RR 1.04, 95% CI 1.02‒1.06) and 13% in the contrast between conventional and non-territorial shared-room offices (RR 1.03, 95% CI 1.01‒1.04).

Table 3 displays descriptive statistics for each of the job control items, overall and by office concept. The tendency was the same across all four items; means were generally highest among employees in private offices and lowest in the non-territorial office types. Inter-item correlations ranged from 0.37 between “decide your own work pace” and “decide how to do your work” to 0.48 between “decide how to do your work” and “influence decisions about your work”. All were statistically significant. Considering the imbalance in conceptual content, we ran regression models with an alternative job control variable that only included the three latter items (α=0.71). Models yielded almost identical results and would not have resulted in any substantively different conclusions.

**Table 3:** Means and standard deviations (SD) of job control items, overall and by office concept.

Item	Overall		Private		Conventional shared-room		Conventional open-plan		Non-territorial shared-room		Non-territorial open-plan
	Mean (SD)		Mean (SD)		Mean (SD)		Mean (SD)		Mean (SD)		Mean (SD)
Decide your own work pace	2.59 (1.02)		2.79 (0.96)		2.50 (1.00)		2.52 (1.01)		2.21 (1.07)		2.23 (1.12)
Decide which tasks you are given	2.12 (1.14)		2.35 (1.14)		2.01 (1.12)		2.05 (1.09)		1.78 (1.12)		1.64 (1.08)
Decide how to do your work	2.82 (1.00)		3.01 (0.92)		2.76 (1.00)		2.78 (1.01)		2.50 (0.99)		2.41 (1.12)
Influence decisions about work	2.59 (0.96)		2.79 (0.93)		2.50 (0.95)		2.49 (0.91)		2.31 (0.96)		2.26 (0.96)

## Discussion

Despite empirical evidence linking shared and open workspaces to unfavorable outcomes such as sickness absence (3–5), few empirical studies have focused on potential mechanisms that contribute to these associations. One salient feature that sets different office concepts apart is psychological privacy. We proposed that limited means to pace and regulate social interaction in shared and open workspaces may influence employees’ job control negatively and that job control therefore represents a potential mediator linking shared and open workspaces to higher sickness absence. We tested this proposition with a mediation model hypothesizing that job control would mediate differences in risk of physician-certified sickness absence between private offices and shared and open workspaces.

We found significantly higher sickness absence risk in conventional open-plan offices and non-territorial shared-room and open-plan offices compared to private offices. While echoing findings from previous studies on office concepts and sickness absence (5), we are the first to examine these associations in a nationally representative sample using physician-certified sickness absence. Save for one study in a non-representative sample (7), all previous studies have relied on either self-reported (6, 8, 9) or employer-reported (10) sickness absence. As registry-based physician-certified sickness absence may differ from other sickness absence measures both methodologically (eg, not self-reported) and empirically (eg, less prevalent, other underlying mechanisms), more studies using this type of outcome data are warranted.

Our findings suggest that job control may be a potential mediator of differences in sickness absence risk across office concepts. We found significant indirect effects through job control in all contrasts, and significant proportions of the higher sickness absence risk were attributable to lower job control in shared and open workspaces than in private offices. Together with previous studies indicating similar differences in job control across office concepts (22, 23), this points to interesting avenues for future research, such as experimental studies to provide a stronger basis for causal inference. Job control is an important psychosocial determinant of employee health and well-being in general (16). Studies investigating its mediating role in associations between office concepts and other employee outcomes is therefore warranted. Although indirect effects were significant, direct effects indicated that most of the differences in sickness absence risk might be due to other pathways. Mechanisms underlying differences in employee health and well-being across office concepts is likely complex. More studies aimed at testing the role of different mediators is therefore needed.

Considering the increasing prevalence of shared and open workspaces without assigned workstations, we differentiated shared and open workspaces based on the territoriality dimension. While all shared and open workspaces save space by reducing average square meter per workstation, non-territorial types enable organizations to save space also by increasing the average number of employees per workstation (29). While a common assumption is that these office types may promote employees’ control over workspace (29), scholars have also pointed to potential caveats of non-territoriality, such as work disruption due to moving around to find appropriate workstations (38). In line with the latter perspective, we found lower ratings of job control also for the non-territorial office types. It is important to note that we were unable to differentiate between non-territorial office types beyond the shared-room/open-plan distinction. Thus, our data did not enable us to identify types of non-territorial open-plan offices explicitly aimed at fostering experiences of control, most importantly activity-based offices (27, 30). Our findings nevertheless suggest that non-territoriality itself does not enhance job control. This interpretation echoes previous studies that suggest non-territoriality seem to limit, rather than enhance, employees’ control over workspace and the facilitation of work task completion (26, 39).

Conventional and non-territorial office types further differed from each other in overall sickness absence risk compared to private offices (ie, total effects). We found significantly higher risk in non-territorial compared to conventional shared-room offices. Risk was also elevated in non-territorial compared to conventional open-plan offices. This is insightful, considering previous findings related to non-territorial office types and sickness absence have been inconsistent (6, 8, 10). Yet, sample characteristics separated by office concept indicated that employees in the non-territorial office types, particularly those in non-territorial shared-room offices, differed most from employees in private offices. A large share of employees in non-territorial shared-room offices spent less than half their time on office work and one third worked in service occupations. Although we adjusted for occupation group and time spent on office work, these characteristics suggest that this office category includes employees that are not typical office workers. One important methodological implication of differences in effects across contrasts is that studies investigating associations between office concepts and employee outcomes should distinguish between territoriality and non-territoriality as well as between shared-room offices and open-plan offices.

### Practical implications

Increasing empirical evidence suggests that shared and open workspaces represent risk factors for sickness absence, but providing all employees with private offices is not a viable option for many organizations. Understanding why differences in sickness absence between private offices and shared and open workspaces occur is therefore important to help identify potential targets for intervention that are practically relevant and feasible for organizations. Based on our findings, seeking ways to enhance job control among employees in shared and open workspaces may represent one such target. This includes helping employees to engage in job crafting to foster workspace control (40), such as providing flexible partition walls and enough opportunities to work in private. When implementing new office concepts, organizations should be aware that employees’ job control might be affected. Yet, small indirect effects suggest that practical implications may be limited or that other factors in the work environment may be equally important in addressing the increased sickness absence risk associated with shared and open workspaces. Furthermore, some employees may never thrive in shared and open workspaces (41). Thus, under certain circumstances, allowing employees to remain in private offices or providing other ways to work in private, could be the best option to ensure a sustainable working life for all.

### Methodological considerations

We used observational data from a large nationally representative sample, merged with physician-certified sickness absence data from national registries. Whereas this implies strong external validity, there are limitations with regards to internal validity. Causal interpretation of results from mediation analysis rests upon assumptions of no unmeasured confounding, which are impossible to verify in observational studies (42). We included multiple baseline confounders to reduce bias. For instance, adjusting for both education level and leadership/executive responsibility closed back-door paths contributing to the association between office concept and job control due to differences in education level as well as differences in leadership/executive responsibility among employees with the same education level. While findings are in line with the proposed data generating process and hypothesized relationships, we cannot rule out alternative explanations and causal interpretations should be made cautiously. Future studies with a stronger basis for causal inference, such as experimental designs, are needed to advance the study of mechanisms underlying observed patterns of sickness absence and other employee outcomes across office concepts.

While the study used prospective registry data on sickness absence, data on office concepts and job control came from the same timepoint, which is not ideal for testing mediation. Although it is less likely that ratings of job control would influence what office concept you are placed in, a better design for mediation analysis would have been to measure the exposure, mediator, and outcome at different timepoints. Survey data from only one timepoint also prohibited us from accounting for potential changes in the variables during the follow-up period and these unmeasured changes could have influenced the results.

Measurement error in the mediator might also have influenced parameter estimates (43). Although scale reliability of our four-item measure was satisfactory, the study would have benefited from a more balanced assessment of different aspects of job control. Future studies should examine the proposed relationships with a psychometrically validated measure of job control, including the potential link between psychological privacy and job control on which our hypotheses are based.

Office concept was self-reported at the individual level and not assessed objectively. Thus, one might argue that the measure more adequately reflects individual workstation situation. The office concept questions might also have been ill-suited for employees in non-territorial offices as the number of people in the same room may vary. These issues introduce a risk of measurement error in the form of misclassification of individuals into office concepts. As this limitation is not unique to this study, it underscores a pressing need for further questionnaire development in research on office concepts.

Unlike several previous studies examining differences in sickness absence across office concepts, we had large sample sizes in the non-territorial office categories. Yet, our data did not enable us to differentiate between different types of shared and open workspaces, particularly activity-based offices, or the presence of features such as quiet work zones meant to address challenges in shared and open workspaces. Survey data also did not include questions regarding telework from home. This is a limitation, as the amount of telework from home, and the possibility to do so, may vary across different office concepts and influence job control and sickness absence/presence. It might also limit the study’s relevance for modern office concepts such as activity-based offices or hybrid work arrangements. Yet, knowledge about how employees are affected by their physical and social environment at work is relevant, regardless of whether employees can work from home (6).

The fact that employees in Norway receive full pay compensation from the first day of sickness absence might limit the generalizability of findings to countries with other compensation schemes.

### Concluding remarks

This study makes an important conceptual and empirical contribution to the study of how physical and social aspects of the office environment potentially influence employee outcomes such as sickness absence. Future research should continue to explore potential mediators, both to advance scientific inquiry into underlying mechanisms and to identify potential targets of evidence-based interventions for practitioners and researchers alike.
